# Clinical features and genetic analysis of acrodermatitis enteropathica in an ethnic minority infant from Western China: a case report and literature review

**DOI:** 10.3389/fmed.2025.1616196

**Published:** 2025-10-01

**Authors:** Tuerhongjiang Axirejiang, Gulinigeer Simayi, Abudushalamu Abuduwake, Yunxia Liu, Gang Zheng, Irshat Ibrahim

**Affiliations:** ^1^The First People’s Hospital of Kashi, Kashi, China; ^2^Army 947 Hospital of the Chinese People’s Liberation Army, Kashgar, China

**Keywords:** acrodermatitis enteropathica, *SLC39A4* gene, zinc deficiency, genetic testing, rare disease

## Abstract

**Background:**

Acrodermatitis enteropathy (AE) is a rare autosomal recessive disorder caused by mutations in the *SLC39A4* gene. It is characterized by acral and perioral dermatitis, alopecia, and diarrhea. We reported the clinical features, genetic findings, and treatment response of a minority ethnic infant with AE from Xinjiang, China, and reviewed advances in AE management.

**Case description:**

A 10-month-old minority female infant presented with characteristic perioral, acral, and perianal erythematous erosions; alopecia; and diarrhea. Her serum zinc level significantly decreased (0.19 mg/L). Whole-exome sequencing revealed a homozygous c.197G > T(p. C66F) mutation in the *SLC39A4* gene. Her skin lesions improved, her hair regrew, and her serum zinc level increased to 0.62 mg/L following zinc supplementation (3 mg/kg/d) and comprehensive treatment.

**Conclusion:**

This is the first case of AE in a Xinjiang minority infant and implicates *SLC39A4* mutations in its pathogenesis and zinc therapy efficacy. This case provides insights into the genetic and clinical features of AE across Chinese ethnic groups, emphasizing early genetic testing and individualized zinc therapy. Primary physicians should consider AEs in infants with characteristic dermatitis, alopecia, and diarrhea. The zinc levels should be measured promptly, and genetic analysis should be conducted to prevent misdiagnosis and treatment delays.

## Introduction

Acrodermatitis enteropathy (AE) is a rare autosomal recessive genetic disorder characterized by dermatitis on the extremities and perioral area, alopecia, and diarrhea (1). Its prognosis has significantly improved due to the widespread use of zinc supplementation (2). The pathogenesis of AE is linked primarily to impaired zinc absorption, especially due to deficiency or dysfunction of the ZIP4 transporter protein in the small intestine (3). The ZIP4 protein is encoded by the *SLC39A4* gene, and mutations in this gene directly cause inadequate zinc absorption in the body, triggering a series of clinical symptoms (4). Over 100 types of *SLC39A4* gene mutations, including missense mutations, nonsense mutations, and splice site variations, have been identified worldwide; these mutations can present in homozygous or compound heterozygous states (5, 6).

Acrodermatitis enteropathy usually manifests during infancy, especially in babies who have been weaned or who did not receive sufficient breast milk (7). Early symptoms typically involve skin lesions, especially erythematous scaling around the perioral area and extremities, progressing to alopecia and diarrhea (8). Although the “triad” of symptoms is considered typical for AE, considerable phenotypic heterogeneity exists in clinical presentation, with certain patients exhibiting mild or incomplete symptoms (9, 10). If untreated, AE can lead to growth retardation, immune dysfunction, and even neurological damage (11).

Although rare, AE is often misdiagnosed or overlooked in clinical practice because its symptoms resemble those of more common skin diseases, such as eczema, atopic dermatitis, and seborrheic dermatitis (8). Genetic testing is a crucial tool for accurate diagnosis of AE, with *SLC39A4* gene screening becoming a key diagnostic method for suspected cases (12).

Only a few reports are available on AE in minority populations in China; studies on cases from minority regions of Xinjiang are even rarer. In the present study, we report a clinical case of a 10-month-old Uyghur infant with AE, detailing the clinical presentation, diagnostic process, and treatment response. In addition, we reviewed the related literature and summarized the clinical experience in the diagnosis and treatment of AE to provide clinicians with precise diagnostic methods and treatment strategies, improve the clinical awareness of AE, and support early intervention.

## Case report

**FIGURE 1:**
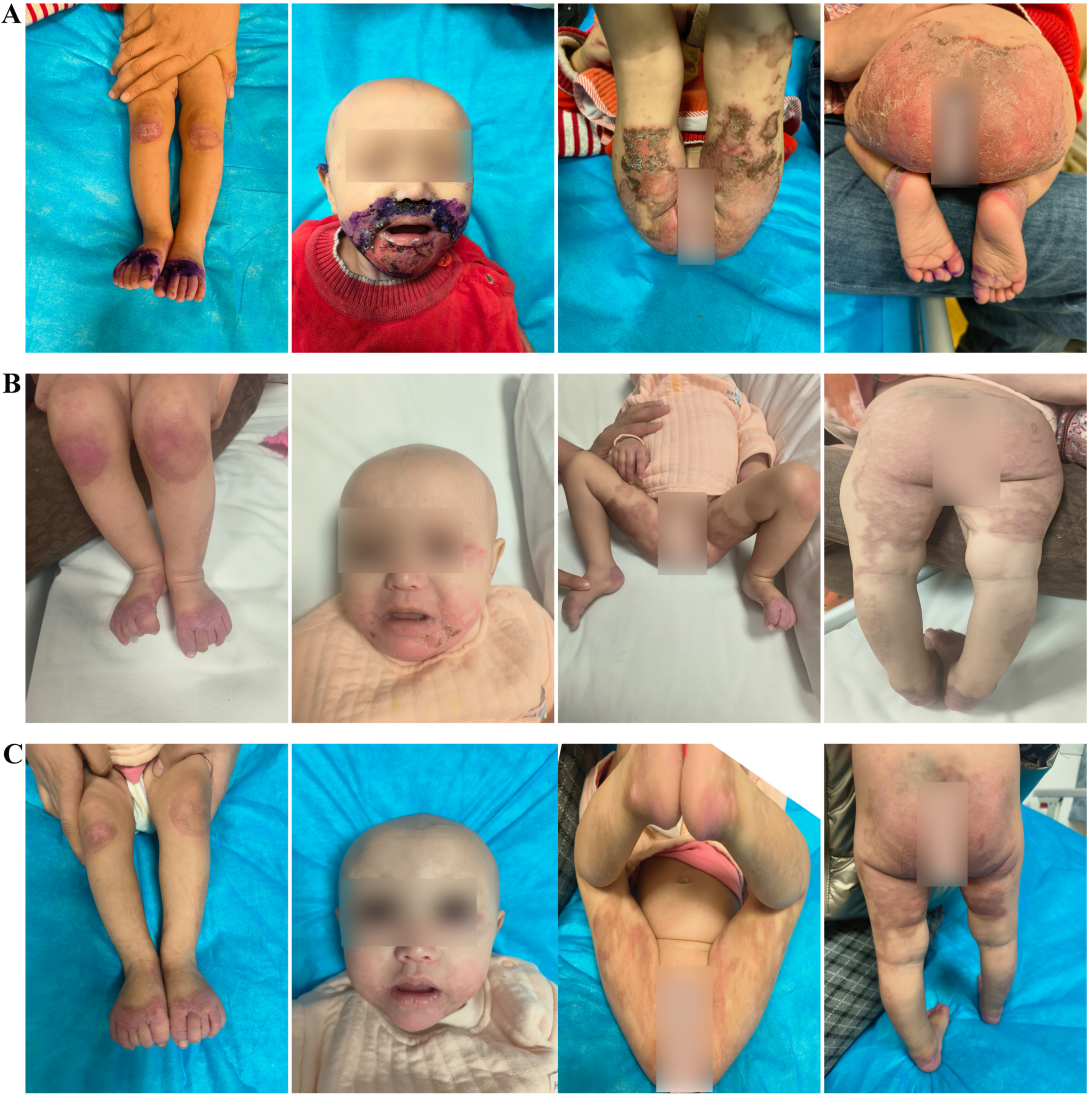
**(A)** Initial widespread erosive rash. **(B)** Healing after 2 weeks of zinc therapy. **(C)** After 4 weeks with normal serum zinc (0.85 mg/L) and hair regrowth.

We report a case of a 10-month-old Uyghur female infant, weighing 6 kg (<3rd percentile), born at term via normal delivery and previously healthy. She was exclusively breastfed until 6 months, after which complementary foods were introduced. The patient presented with “erythema and erosion on the extremities and perioral and perianal areas, along with alopecia for 2 months and diarrhea for 1 month”. The patient presented to the outpatient department and was admitted with a diagnosis of “staphylococcal scalded skin syndrome”. The skin lesions initially appeared on the left knee and later spread symmetrically to the perioral area, periorbital region, behind the ears, distal extremities, and perianal area, presenting as well-defined erythema, erosion, and crusting (Figure 1A). Physical examination revealed poor general condition, with ring-shaped erythema with crusting around the mouth, prominent erythema and scaling at the extremities, and sparse hair on the scalp. Laboratory tests revealed a hemoglobin level of 84 g/L, a white blood cell count of 16.63 × 10^9^/L, a platelet count of 475 × 10^9^/L, an albumin level of 34.4 g/L, and a significantly low serum zinc level of 0.19 mg/L (reference range: 0.76–1.50 mg/L). The culture of the skin lesion secretions revealed a small number of gram-positive rods. Considering the clinical presentation of dermatitis, alopecia, and diarrhea alongside laboratory evidence of severe hypozincemia, acrodermatitis enteropathica (AE) was suspected. Differential diagnoses initially included staphylococcal scalded skin syndrome (SSSS), atopic dermatitis with secondary infection, and nutritional dermatitis. However, the chronicity, distribution of lesions, and poor response to initial antibiotic therapy argue against SSSS. Whole-exome sequencing revealed a homozygous mutation in the *SLC39A4* gene, c.197G > T (p.C66F), with both parents being heterozygous carriers of this mutation. We diagnosed the patient with acrodermatitis enteritis, secondary skin infection, malnutrition, and moderate anemia on the basis of the typical clinical triad, significantly low serum zinc levels, and genetic test results (Table 1). The treatment included high-dose zinc replacement (elemental zinc 3 mg/kg/d), local anti-infection and anti-inflammatory therapy, and nutritional support. Skin lesions significantly improved, and the serum zinc level increased to 0.62 mg/L after 2 weeks of treatment. The skin lesions nearly completely resolved (Figure 1B), hair regrowth began, diarrhea symptoms improved, and the serum zinc level increased to 0.85 mg/L after 4 weeks (Figure 1C). The zinc dosage was subsequently adjusted to a maintenance level (1–2 mg/kg/d), and lifelong zinc replacement therapy was emphasized as necessary.

**TABLE 1:** Laboratory test results upon admission.

Factor	Result	Reference range
Zinc (Zn)	0.19 mg/L	0.76–1.50
Iron (Fe)	3.50 μmol/L	5.2–29.4 μmol/L
Culture result	Negative	–
Alanine aminotransferase (ALT)	8.71 U/L	–
Aspartate aminotransferase (AST)	36.0 U/L	–
Total bilirubin (TBIL)	3.30 μmol/L	3.4–19 μmol/L
Direct bilirubin (DBIL)	0.60 μmol/L	0–6.8 μmol/L
Indirect bilirubin (IBIL)	2.70 μmol/L	5.1–12.2 μmol/L
Total protein (TP)	64.30 g/L	65–85 g/L
Albumin (ALB)	34.40 g/L	40–55 g/L
Globulin (GLB)	29.90 g/L	20–40 g/L
Albumin/globulin ratio (A/G)	1.15	1.2–2.4
Total bile acid (TBA)	11.4 μmol/L	<10 μmol/L
Alkaline phosphatase (ALP)	66.00 U/L	400–1500 U/L
γ - glutamyl transferase (γ - GT)	15.00 U/L	7–32 U/L
Urea	2.71 mmol/L	2.9–8.2 mmol/L
Creatinine (Crea)	18.5 μmol/L	53–97 μmol/L
Uric acid (UA)	214.0 μmol/L	155–357 μmol/L
Potassium (K)	4.83 mmol/L	3.5–5.5 mmol/L
Sodium (Na)	139.0 mmol/L	136–146 mmol/L
Chloride (CL)	103.80 mmol/L	99–110 mmol/L
Calcium (Ca)	2.40 mmol/L	2.2–2.8 mmol/L
Inorganic phosphorus (P)	1.63 mmol/L	1.29–2.26 mmol/L
Magnesium (Mg)	0.99 mmol/L	0.5–0.9 mmol/L
Reticulocyte count (RET)	0.1714 × 10^12^/L	0.0200 × 10^12^/L
Reticulocyte percentage (RET%)	4.72%	0.30%–3.00%
Immature reticulocyte fraction (IR)	17.60%	3.1%–14.4%
Low - fluorescence reticulocyte percentage (LFR)	82.40%	87%–98.5%
Medium - fluorescence reticulocyte percentage (MFR)	13.50%	2.8%–11.8%
High - fluorescence reticulocyte percentage (HFR)	4.00%	0.1%–1.5%
Ferritin	21.98 ng/ml	4.63–204.00 ng/ml
Folate	20.7 nmol/L	7.0–46.4 nmol/L
Vitamin B12 (VB12)	239 pmol/L	138–652 pmol/L
Red blood cell (RBC)	4.05 × 10^12^/L	3.8–5.1 × 10^12^/L
Hemoglobin (HGB)	84.0 g/L	97–141 g/L
Platelet (PLT)	912 × 10^9^/L	117–144 × 10^9^/L
White blood cell (WBC)	16.63 × 10^9^/L	4.8–14.6 × 10^9^/L
D - dimer	0.51 mg/L	0–0.55 mg/L
Fibrin (ogen) degradation product (FDP)	2.50 μg/ml	0–5 μg/ml
Antithrombin III (AT - III)	78.90%	75%–125%
Interleukin 6 (IL6)	32.66 pg/ml	<7 pg/ml
Human immunodeficiency virus antigen and antibody	0.07 S/CO	0–1 S/CO
Treponema pallidum antibody (Anti - TP)	0.58 S/CO	0–1 S/CO

## Literature review

### Genetics and molecular pathology research

Acrodermatitis enteropathica (AE) is a rare genetic disorder caused by mutations in the *SLC39A4* gene, which is located in the 8q24.3 chromosomal region; this gene encodes the ZIP4 zinc transporter protein (4). ZIP4, a type II transmembrane protein comprising eight transmembrane domains, is expressed predominantly in the epithelial cells of small intestinal villi, where it plays a key role in mediating zinc absorption from the intestinal lumen into epithelial cells (3). To date, over 100 mutations of the SLC39A4 gene have been reported globally, including missense mutations (48%), nonsense mutations (17%), and splice site variations (15%), as well as frameshift mutations, large deletions, and insertions (1, 13).

Recent studies have reported that different *SLC39A4* mutation sites may cause varying degrees of functional impairment in the ZIP4 protein, consequently affecting the severity of clinical phenotypes and treatment response (6). For example, mutations located in the transmembrane domains of a protein usually cause more severe zinc absorption defects, whereas those in intracellular or extracellular regions may retain certain protein functions (14). Furthermore, some compound heterozygous mutations can result in distinct phenotypic characteristics due to interactions between mutation sites (15). The c.926G > T and c.1124T > C mutations identified in this case are located within the transmembrane domains of the ZIP4 protein, which may significantly impact both the protein’s structure and function, aligning with the patient’s typical clinical presentation.

Genetic testing has emerged as the gold standard for diagnosing AE, especially in cases with atypical clinical presentations or when differential diagnosis with other skin diseases is challenging (2). Furthermore, whole-exome sequencing can reveal novel pathogenic variations, expanding the mutation spectrum of the *SLC39A4* gene and laying the groundwork for genotype-phenotype correlation studies (16).

### Clinical features and phenotypic diversity

The typical clinical presentation of AE is the “triad,” that is, perioral and extremity dermatitis, alopecia, and diarrhea (17). However, significant phenotypic heterogeneity has been reported clinically in certain patients presenting only mild skin lesions or incomplete combinations of clinical symptoms (8, 18). For example, a multicenter study involving 29 patients with AE reported that skin lesions (97%) were the most common, whereas alopecia (82%) and diarrhea (63%) were less common (5). Skin damage is symmetric with well-defined erythema, blisters, erosions, and crusting, primarily affecting the perioral area, perianal area, fingertip (toes), and extensor joints, often accompanied by secondary infections (19).

The clinical manifestations of AE are influenced by multiple factors, such as age, ethnicity, environmental factors, and genetic background (20). AE in neonates is more acute and severe than that in other age groups and is often accompanied by systemic inflammatory responses, whereas AE in older children may manifest as chronic and recurrent skin lesions, with milder systemic symptoms (1). In addition, AEs may exhibit clinical differences among different ethnic groups, possibly linked to regional dietary habits, environmental factors, and genetic backgrounds (5). The AE cases reported in the present study provide new data for exploring the clinical features of this disease in different ethnic populations.

### Physiological function of zinc and **the** pathophysiology of zinc deficiency

Zinc, an essential trace element, has been implicated in more than 300 enzymatic activities and structural maintenance, influencing processes such as cell proliferation, differentiation, DNA synthesis and repair, protein synthesis, immune function, and antioxidant defense (21, 22). Zinc is involved in the differentiation and proliferation of keratinocytes in the skin, maintaining epidermal barrier integrity and modulating inflammatory responses by regulating the expression of inflammatory cytokines (23, 24).

Zinc deficiency-induced skin lesions involve the following mechanisms: (1) impaired keratinocyte differentiation causing abnormal stratum corneum function; (2) decreased basal cell proliferation, affecting skin renewal; (3) delayed wound healing; (4) enhanced inflammatory responses; and (5) increased cell apoptosis (25, 26). These pathological changes ultimately cause the characteristic skin and mucosal damage observed in patients with AE. In addition, zinc deficiency also impacts immune system function, causing T-cell and B-cell dysfunction and consequently increasing the risk of infections, potentially explaining the common occurrence of secondary skin infections in these patients (27).

### Differentiating AE from acquired zinc deficiency

Congenital AE differs clinically from acquired zinc deficiency syndrome. Acquired zinc deficiency is often caused by malnutrition, malabsorption diseases (such as Crohn’s disease, short bowel syndrome, and celiac disease), or certain medications (such as proton pump inhibitors and oral contraceptives) (28). Although both conditions have similar clinical presentations, acquired zinc deficiency usually has a later onset and is related to the primary disease or its triggers, with no clear family history (29).

Genetic testing is the definitive method for diagnosis. In our case, whole-exome sequencing identified compound heterozygous mutations in the *SLC39A4* gene, confirming congenital AE. In contrast, symptoms are completely resolved with zinc supplementation in patients with acquired zinc deficiency, whereas patients with congenital mutations require lifelong zinc supplementation.

## Discussion

### Ethnic specificity and region-related factors

This study reports the first case of an AE infant from the Uyghur ethnic group in Kashgar, Xinjiang, China. These findings provide valuable data for studying the genetic and clinical characteristics of AE in different ethnic populations. Uyghurs, one of the major minority ethnic groups in China, possess unique genetic backgrounds and lifestyles that may affect the onset and clinical presentation of AE (30).

**FIGURE 2:**
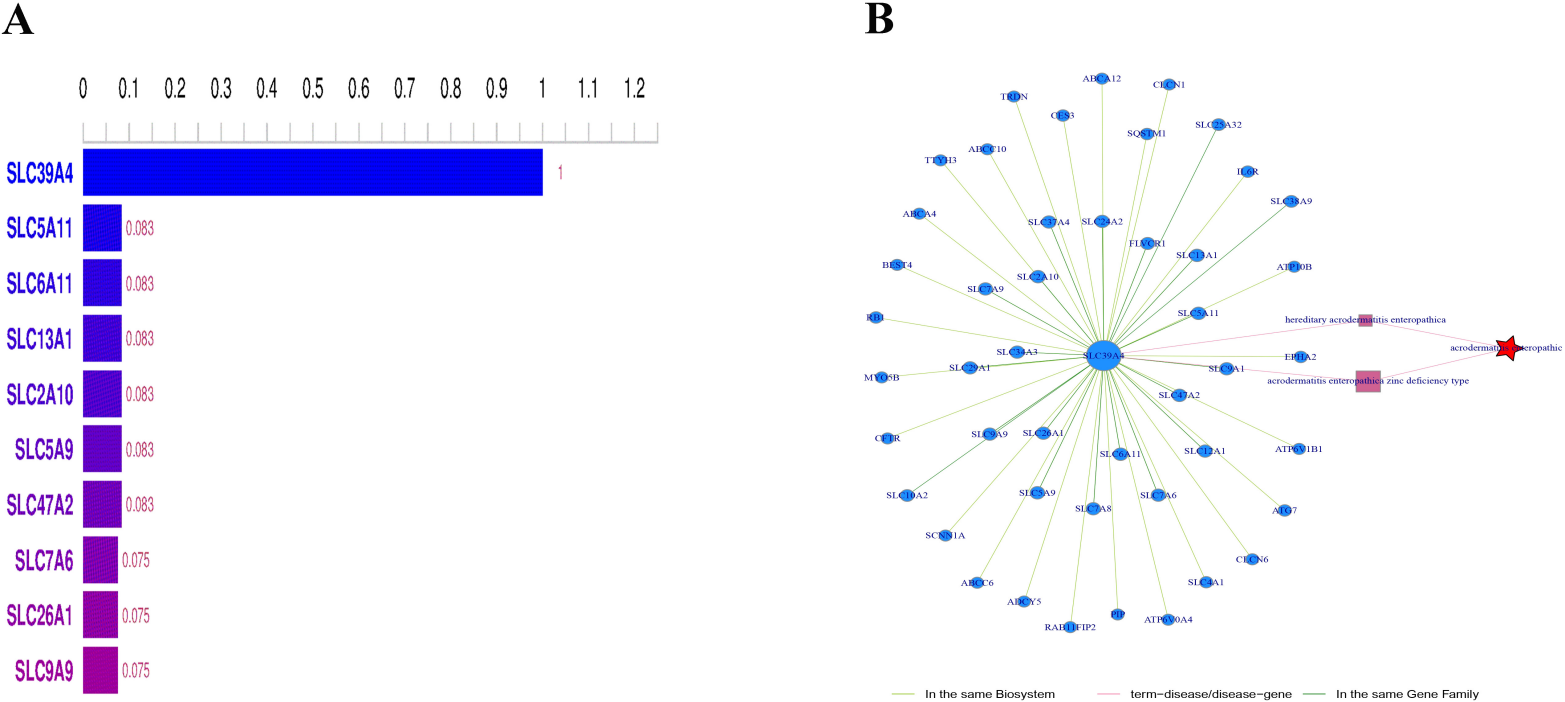
**(A)** SLC39A4 homozygous c.197G>T (p.C66F) mutation. **(B)** Parents heterozygous.

Research has demonstrated that the mutation spectrum of the *SLC39A4* gene varies among different ethnic and regional populations. For example, homozygous mutations in the *SLC39A4* gene are more common in consanguineous families in the Middle East, whereas compound heterozygous mutations predominate in European and American populations (4). Exome sequencing revealed a homozygous c.197G > T (p.C66F) mutation in the *SLC39A4* gene, which is located in exon 2. Both parents were heterozygous carriers of this mutation, which is consistent with the autosomal recessive inheritance pattern (Figure 2). This may represent a unique genetic variation specific to the Uyghur population.

Moreover, the clinical presentation of AE is affected by regional dietary patterns and nutritional status. Although the traditional diet in Xinjiang is characterized by high protein and fat contents, it involves relatively low intake of plant-based foods, particularly whole grains and legumes (31). Interestingly, the traditional diet in this region, characterized by high intake of animal protein and relatively low consumption of phytate-rich foods such as whole grains and legumes, is known to increase zinc bioavailability. We speculate that this dietary pattern might act as a modifying factor, potentially delaying the onset or mitigating the severity of clinical symptoms in individuals with this genetic predisposition to AE. However, further investigation is needed to confirm this hypothesis.

### Optimizing zinc therapy

Zinc supplementation forms the cornerstone of AE treatment. The recommended dose of elemental zinc varies from 1 to 3 mg/kg/d; it can be individualized on the basis of a patient’s clinical response and serum zinc level (32). In our case, we initially used a higher dose (3 mg/kg/d) of elemental zinc, which gradually reduced the maintenance level (1–2 mg/kg/d) as the patient’s symptoms improved and serum zinc levels increased. This “high start, gradual taper” strategy assists in rapidly relieving symptoms while preventing zinc overdose. Different zinc formulations (such as zinc sulfate, zinc gluconate, and zinc acetate) have been reported to have varying bioavailabilities and adverse effects (33). Zinc gluconate is often the preferred formulation, particularly for infant patients, owing to its good gastrointestinal tolerance and absorption rate (34). In addition, treatment response should be monitored via different indicators, such as serum zinc levels reflecting short-term zinc status, whereas alkaline phosphatase activity, skin lesion healing, and hair regrowth serve as long-term indicators of zinc status (35, 36).

Importantly, long-term zinc supplementation may affect the absorption of other trace elements, such as copper and iron. Therefore, regular monitoring of copper and iron status is recommended for patients with AE receiving lifelong zinc treatment, with appropriate interventions, if necessary (37).

### Gene-environment interactions

The clinical presentation of AE is not affected by genetic or environmental factors. In our case, the infant was exclusively breastfed during the first 6 months of life, and symptoms began to appear after complementary foods were introduced, which is consistent with the related literature (38). Compared with formula milk and complementary foods, breast milk, particularly colostrum, contains relatively high zinc levels and bioavailability, which may temporarily mask zinc absorption defects caused by ZIP4 dysfunction (39). Other environmental factors that may influence AE phenotypes include (1) maternal zinc status, which can affect the zinc content of breast milk and potentially accelerate the onset of AE symptoms (40); (2) the gut microbiome, which may impact zinc bioavailability and absorption; (3) concurrent infections or inflammation, which can increase zinc demand and utilization (41); and (4) the dietary presence of phytates and fiber, which can chelate zinc ions and reduce absorption (42). These findings emphasize the need for a holistic approach to managing patients with AE, considering not only genetic mutations and zinc supplementation but also nutrition, gut health, and environmental factors.

### Genetic mutation characteristics and functional implications

The SLC39A4 c.197G > T (p.C66F) mutation identified in this case is located in the extracellular N-terminal domain of the ZIP4 protein, a region highly conserved within ZIP4 but only partially conserved among other ZIP family members. This residue is likely crucial for maintaining protein structural stability through disulfide bond formation. Structural prediction analyses indicate that this mutation does not reside within any of the eight transmembrane domains, suggesting that its impact on zinc transport is indirect, likely mediated by the disruption of disulfide bonds or local conformational changes that affect protein stability and zinc binding efficacy. Notably, this variant has not been reported in major mutation databases or the literature, indicating that it is a novel pathogenic mutation that is potentially specific to the Uyghur population or the Xinjiang region. Clinically, this mutation corresponds with the classic acrodermatitis enteropathica phenotype, and the patient showed a good response to zinc supplementation, supporting residual ZIP4 function. These findings enrich the mutation spectrum of SLC39A4 and underscore the importance of the detailed characterization of rare mutations for understanding disease mechanisms and guiding personalized therapy.

### Raising awareness among physicians in remote areas

Our patient was initially misdiagnosed with “staphylococcal scalded skin syndrome,” leading to a delay in appropriate treatment. This highlights the urgent need to raise awareness of rare diseases among primary care physicians in remote areas (43).

Three strategies are recommended to address diagnostic challenges for AE in remote areas: first, the development of simplified clinical screening tools, utilizing “perioral/extremity dermatitis, alopecia, diarrhea and growth retardation” as key screening indicators to identify suspected cases at primary care clinics; second, the establishment of regional graded diagnosis and treatment networks, offering teleconsultation for primary care physicians, enabling timely referrals to higher-level hospitals, and ensuring the availability of serum zinc level tests; and third, the creation of standardized diagnostic and treatment protocols for AE and other rare diseases, along with training courses. These training programs should be integrated into the continuing education system for primary care physicians, incorporating case discussions, pattern recognition exercises, and simplified treatment guidelines to increase awareness of rare diseases.

### Strengths

This study is the first to report a novel SLC39A4 gene mutation, c.197G > T (p.C66F), that causes acrodermatitis enteropathica (AE) in an infant of the Uyghur ethnic group, thereby expanding the mutation spectrum. The diagnosis was confirmed by whole-exome sequencing, and zinc supplementation led to significant clinical improvement, validating the clinical utility of genetic diagnosis. Considering the unique dietary background of the Uyghur population, this study preliminarily explored gene-environment interactions.

### Limitations

This study is limited by the use of a single case, which limits the generalizability of the conclusions. The levels of other transition metals before and after treatment were not measured, and the dietary assessment lacked quantitative analysis. The functional impact of the p.C66F mutation was inferred solely from bioinformatic predictions without experimental validation.

## Conclusion

This study reports the first case of AE in a Uyghur infant from Kashgar, Xinjiang, China. This study provides valuable clinical data on the disease’s presentation and management. This case highlights the importance of genetic testing in the diagnosis of AE and demonstrates the effectiveness of zinc supplementation in its treatment. Early recognition, accurate diagnosis, and individualized treatment can lead to favorable outcomes for most patients with AE. Future research should focus on exploring the genetic diversity, phenotypic heterogeneity, and precision treatment strategies for AE to improve global diagnostic and therapeutic levels for this rare disease.

## Data Availability

The datasets presented in this study can be found in online repositories. The names of the repository/repositories and accession number(s) can be found in this article/Supplementary material.
